# Effectiveness and tolerance of single tablet versus once daily multiple tablet regimens as first-line antiretroviral therapy - Results from a large french multicenter cohort study

**DOI:** 10.1371/journal.pone.0170661

**Published:** 2017-02-02

**Authors:** Laurent Cotte, Tristan Ferry, Pascal Pugliese, Marc-Antoine Valantin, Clotilde Allavena, André Cabié, Isabelle Poizot-Martin, David Rey, Claudine Duvivier, Antoine Cheret, Pierre Dellamonica, Pierre Pradat, Jean-Jacques Parienti, Dat’AIDS study group

**Affiliations:** 1 Department of Infectious Diseases, Croix-Rousse Hospital, Hospices Civils de Lyon, Lyon, France; 2 INSERM U1052, Lyon, France; 3 Department of Infectious Diseases, Centre Hospitalier Universitaire de Nice, Hôpital l’Archet, Nice, France; 4 Department of Infectious Diseases, Assistance Publique - Hôpitaux de Paris, Pitié-Salpêtrière Hospital, Paris, France; 5 Sorbonne University, UPMC University, Paris, France; 6 INSERM, Institut Pierre Louis d’épidémiologie et de Santé Publique (IPLESP UMRS 1136), Paris, France; 7 Department of Infectious Diseases, Hôtel Dieu, Nantes, France; 8 Department of Infectious Diseases, CHU de Martinique, Fort-de-France, France; 9 Université des Antilles EA4537, Fort-de-France, France; 10 INSERM CIC1424, Fort-de-France, France; 11 Immuno-hematology clinic, Assistance Publique – Hôpitaux de Marseille, Hôpital Sainte-Marguerite, Marseille, France; 12 Aix-Marseille University, Marseille, France; 13 INSERM U912 (SESSTIM), Marseille, France; 14 HIV Infection Care Centre, Hôpitaux Universitaires, Strasbourg, France; 15 Department of Infectious Diseases, Centre d’Infectiologie Necker-Pasteur, IHU Imagine, Assistance Publique - Hôpitaux de Paris, Hôpital Necker-Enfants Malades, Paris, France; 16 Université Paris Descartes, Sorbonne Paris Cité, EA7327, Paris, France; 17 Department of Internal Medicine, Centre Hospitalier Universitaire, Bicètre, France; 18 Center for Clinical Research, Department of Hepatology, Croix-Rousse Hospital, Hospices Civils de Lyon, Lyon, France; 19 Department of Infectious Diseases, Côte de Nacre University Hospital, Caen, France; 20 Department of Biostatistics, Côte de Nacre University Hospital, Caen, France; National and Kapodistrian University of Athens, GREECE

## Abstract

**Objectives:**

Pill burden during antiretroviral treatment (ART) is associated with worse adherence and impaired virological suppression. We compared the effectiveness, tolerance, and persistence on treatment of single tablet regimens (STRs) with non-STR once-daily regimens in patients receiving first-line ART.

**Methods:**

Retrospective analysis of naïve HIV-1 infected patients prospectively enrolled in the French Dat’AIDS cohort and initiating first-line ART with STRs or once-daily non-STRs from 2004 to 2013. The primary outcome was time to treatment discontinuation defined by any change in the treatment regimen. STR and non-STR groups were compared controlling for baseline risk factors by inverse probability weighted treatment Cox analysis (IPWT) and propensity-score matching (PSM).

**Results:**

Overall, 3212 patients (STR 499, non-STR 2713) were included. Median time to treatment discontinuation was shorter in non-STR patients than in STR patients, both in the IPWT (HR = 0.61, p<0.0001) and the PSM cohort (HR = 0.55, p<0.0001). This difference disappeared when censoring ART modification for simplification, both in the IPWT (HR = 0.97, p = 0.65) and the PSM cohort (HR = 0.91, p = 0.33). A lower rate of virological failure was observed with STRs than with non-STRs in both cohorts (HR = 0.23; p = 0.002 and HR = 0.22, p = 0.003, respectively). A lower rate of treatment modification for adverse event was observed with non-STRs in the IPWT cohort (HR = 1.46, p<0.0001), but not in the PSM cohort (HR = 1.22, p = 0.11).

**Conclusion:**

First-line therapy with STRs was associated with a longer time to treatment discontinuation than with non-STRs. However, when ART modification for simplification was not considered as a failure, STRs and non-STRs were similar.

## Introduction

During the past decades, HIV combined antiretroviral therapy (ART) has drastically evolved with as a consequence a better control of HIV infection and a reduction of HIV-related morbidity and mortality. Adherence to treatment has been reported as a key factor for ART success and international guidelines underscore the importance of simplifying regimens to improve adherence [1]. Treatment simplification first became possible with the development of drugs with longer half-lives hence allowing once-daily dosing. Adherence to these once-daily regimens was shown to be significantly better than with 3-times and 4-times-daily regimens [2]. However, in a meta-analysis, Parienti et al. reported only a modest beneficial effect of once-daily regimens on adherence to treatment compared with twice-daily regimens [3]. In 2006, the first single tablet regimen (STR), a combination of tenofovir (TDF), emtricitabine (FTC), and efavirenz (EFV) became available and recommended in first-line therapy [4–6]. Several other STRs combining TDF/FTC/rilpivirine (RPV), TDF/FTC/cobicistat/elvitegravir and abacavir/lamivudine/dolutegravir have been developed [7,8] and as recommended by international guidelines, STRs appear now as a valid treatment strategy to decrease pill burden [1,9]. Compared with multiple tablet regimens, some STRs have been associated with better adherence [10–15], and improved quality of life (QoL) [15,16]. Moreover, since adherence to treatment is correlated with hospitalization, some studies showed that STRs were also associated with a 17% reduction in total health care costs mostly due to a reduction in hospitalizations [12,17]. Recently, some authors described the different factors to be considered for the choice of a particular STR and stressed the need for careful clinical, virological and immunological monitoring along with regular adherence assessment to achieve treatment success [18].

Since comparisons between STRs and non-STR once-daily regimens are sparse, we compared the effectiveness, tolerance, and persistence on treatment of STRs with non-STR regimens in patients receiving first-line ART in a French large cohort of HIV-infected patients.

## Materials and methods

### Patients

All adult naive HIV-1 infected patients prospectively enrolled in the French Dat’AIDS cohort and receiving either an STR or non-STR once-daily first-line ART from 2004 to 2013 with HIV-RNA data >6 months were retrospectively analyzed. The Dat’AIDS cohort represents a collaboration between about 30 major French HIV treatment centers throughout the country [19] and includes data on more than 35,000 HIV-infected patients. Patients were followed until occurrence of one of the following events: treatment modification, treatment interruption, death, lost to follow-up, or end of study period (December 31, 2013).

### Outcomes

The primary outcome was time to treatment discontinuation defined as the delay between starting and stopping the first line antiretroviral therapy. Reasons for stopping therapy which were prospectively collected included simplification, clinical or biological toxicity, virological failure and other reasons such as pregnancy, pharmacological interactions, or patient’s willingness.

Virological failure definition was similar as that used in the ACTG 5202 study [20] and was characterized by a viral load (VL) ≥ 1000 copies/mL between W16 and W24 or VL ≥ 200 copies/mL after W24. Adverse events not leading to treatment modification were not considered as failure. Simplification was defined as any change of the primary treatment resulting in a lower pill burden. For example, a switch from TDF/FTC/EFV as separate pills to TDF/FTC/EFV as STR was considered as simplification. Treatment was considered interrupted after four weeks of discontinuation.

Four different analyses were conducted: (i) Overall effectiveness: In the main analysis, only patients who remained on the same therapy at the end of follow-up were considered as treatment success. The primary outcome was defined as treatment discontinuation for virological failure, occurrence of adverse event, or any cause of treatment modification. (ii) Overall effectiveness, simplification censored: Same as (i) but treatment simplification was censored. (iii) Virological efficacy: Only virological failure was considered as treatment failure. (iv) Tolerance: Only toxicity requiring treatment modification was considered as treatment failure.

### Statistical analysis

Data were expressed as mean ± standard deviation, or median (interquartile range) for quantitative variables or as numbers and percentages for qualitative variables. Baseline differences between patients starting with a non-STR once-daily regimen or an STR were compared using Chi-square or Fisher exact tests for categorical variables and two-tailed, unpaired t test or Wilcoxon rank sum test for continuous variables in the overall cohort. In addition, we compared baseline characteristics by the use of standardized difference.

The association between each endpoint and treatment strategy was evaluated by the hazard ratio (HR) and its 95% confidence interval (CI), with a value <1 being in favor of STR (increased risk of outcome in the non-STR group). Because non-STR and STR were selected by choice rather than by chance, an analysis using propensity scores was conducted to limit potential biases. The propensity score was computed from a logistic regression non-parsimonious model including all baseline characteristics associated with STR with a p<0.5, i.e. age, gender, HIV RNA, CD4 cell count, CDC stage, hepatitis B or C coinfection, HIV risk factor, and type of ART. Two different complementary approaches were used: inverse probability weighting (IPWT) and matching on the propensity score [21]. Each endpoint was modeled by a marginal structural Cox model (IPWT), after checking the proportionality assumption, which was met. Second, a one-to-one greedy 5 to 1 digit technique was performed to match one control (non-STR group) with one case (STR group), based on the propensity score and nested within the overall cohort. In this matched subsample, baseline characteristics included in the propensity score were compared between cases and controls by the standardized difference, as appropriate. The probability of outcome was then modeled in a Cox model with robust sandwich variance estimators to take into account the correlation within each matched pair and included the group (non-STR versus STR) as an explanatory factor. In order to assess the factors associated with the prescription of STR and account for the indication bias in the analyses, we preferred a strategy using propensity score rather than covariable adjustment. A P value <0.05 was considered significant and all P values were two-tailed. No adjustment was made for multiple comparisons. Statistical analyses were performed using SAS statistical software, version 9.4 (SAS Institute Inc., Cary, NC).

### Ethical considerations

The Dat’AIDS cohort received approval from the French “Comité consultatif sur le traitement de l'information en matière de recherche dans le domaine de la santé'” (Registration number 15.196) and is registered with identifier NCT02898987 in ClinicalTrial.gov. All patients included in this study gave their written inform consent to allow the use of their personal clinical data. All patient information was entered into a database using anonymous coded identification numbers. This study was conducted in accordance with the principles of the declaration of Helsinki and the French law related to biomedical research.

## Results

### Patients’ characteristics

Between 2004 and 2013, 3212 patients fulfilling the inclusion criteria were analyzed among whom 499 were treated with STRs, and 2713 with once-daily non-STRs. Patients’ characteristics for the whole cohort and after propensity matching are summarized in Table 1. STR patients were more frequently younger (37.6 vs 39.6 years, p = 0.0002), male (83.4% vs 77.0%, p = 0.002), homo- or bisexual (59.2% vs 48.4%, p<0.0001), had less frequently HBV/HCV coinfection (7.2% vs 10.8%, p = 0.016), were less frequently at CDC stage C (6.0% vs 14.7%) and had higher baseline CD4 level (395/mm3 vs 315/mm3, p<0.0001) and lower baseline HIV-RNA level (4.6 log copies/mL vs 4.7 log copies/mL, p = 0.025) than non-STR patients. A center effect was also observed. Detailed antiretroviral treatment among patients receiving non-STR or STR is presented in S1 Table. Using the propensity score, 487 STR patients were matched with 487 non-STR patients leading to comparable groups (all standardized differences <0.05), except for the type of ART received which was not possible.

**Table 1:** Patients’ characteristics at treatment initiation.

	Overall cohort				After Propensity matching		
	Non-STR (n = 2713)	STR (n = 499)	p	Standardized difference	Non-STR (n = 487)	STR (n = 487)	Standardized difference
Age, mean (SD)	39.6 (11.0)	37.6 (10.4)	0.0002	0.19	37.6 (10.2)	37.7 (10.4)	-0.01
Male, n (%)	2089 (77.0%)	416 (83.4%)	0.0016	-0.16	402 (82.6%)	405 (83.2%)	-0.02
HIV RNA, (log_10_), mean (SD)	4.7 (0.84)	4.6 (0.77)	0.025	0.12	4.6 (0.84)	4.6 (0.77)	0
CD4, mean (SD)	314.9 (183.8)	394.6 (203.4)	<0.0001	-0.41	391.9 (227.4)	390.9 (201.8)	0.005
CDC stage C, n (%)	398 (14.7%)	30 (6.0%)	<0.0001	0.29	26 (5.3%)	30 (6.2%)	-0.04
Hepatitis C or B, n (%)	293 (10.8%)	36 (7.2%)	0.016	0.13	40 (8.2%)	36 (7.4%)	0.03
Risk factors, n(%)			<0.0001				
Heterosexual	1135 (41.8%)	157 (31.5%)		0.21	162 (33.3%)	156 (32.0%)	0.03
Homo & Bisexual	1314 (48.4%)	295 (59.2%)		-0.22	276 (57.3%)	288 (59.1%)	-0.04
Other	264 (9.7%)	47 (9.4%)		0.01	46 (9.5%)	43 (8.8%)	0.02
Type of ART, n(%)			<0.0001				
2 NRTI + 1 NNRTI	872 (32.1)	499 (100%)			102 (20.9%)	487 (100%)	NA
2 NRTI + 1 bPI	1841 (67.9)	0 (0%)			385 (79.1%)	0 (0%)	NA

ART, antiretroviral therapy; NRTI, nucleoside reverse transcriptase inhibitor; NNRTI, non-nucleoside reverse transcriptase inhibitor; bPI, boosted protease inhibitor; SD, standard deviation; STR, single tablet regimen

Overall, the median [Interquartile Range] time before the primary outcome was 1.18 years [0.44–2.26], corresponding to 1.15 years [0.41–2.26] in the non-STR group and 1.34 years [0.59–2.28] in the STR group. During follow-up, 58% of patients receiving STRs had no treatment modification compared with 32% of patients receiving non-STR regimens (p<0.0001, Table 2).

**Table 2:** Primary reason for treatment modification during follow-up.

	Overall cohort			After Propensity matching		
	Non-STR (n = 2713)	STR (n = 499)	p	Non-STR (n = 487)	STR (n = 487)	p
Patients without treatment modification	878 (32.4%)	291 (58.3%)	<0.001	181 (37.2%)	282 (57.9%)	<0.001
Reason for treatment modification, n (%)						
Adverse event	553 (20.4%)	156 (31.3%)	<0.001	101 (20.7%)	154 (31.6%)	<0.001
Virological failure	154 (5.7%)	10 (2.0%)	<0.001	20 (4.1%)	10 (2.1%)	0.0951
Simplification	723 (26.6%)	0 (0%)	-	126 (25.9%)	0 (0%)	-
Other	405 (14.9%)	42 (8.4%)	<0.001	59 (12.1%)	41 (8.4%)	0.072

STR, single tablet regimen; Other includes: pregnancy (planned or ongoing), patient willingness, poor adherence, drug-drug interaction, enrollment in clinical trial, toxicity prevention such as switch from didanosine or stavudine to other drugs when the toxicities of these molecules were widely recognized.

### Treatment discontinuation over time

**Fig 1:**
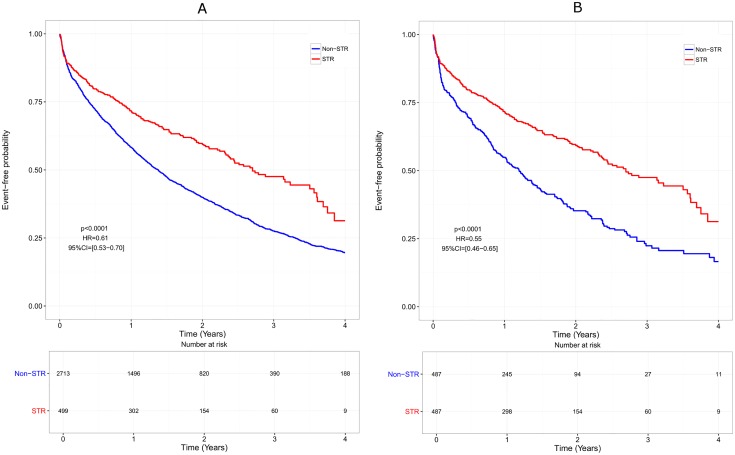
Overall effectiveness over time. Only patients remaining on the same therapy at the end of follow-up are considered as treatment success. Failure is defined as treatment discontinuation, occurrence of adverse event, or any cause of treatment modification.

**Fig 2:**
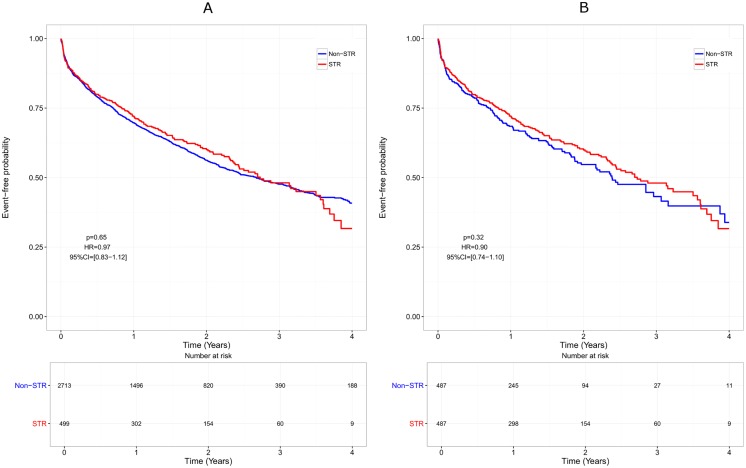
Overall effectiveness over time (simplification censored). Only patients remaining on the same therapy at the end of follow-up are considered as treatment success. Failure is defined as treatment discontinuation, occurrence of adverse event, or any cause of treatment modification except treatment simplification (censored).

**Fig 3:**
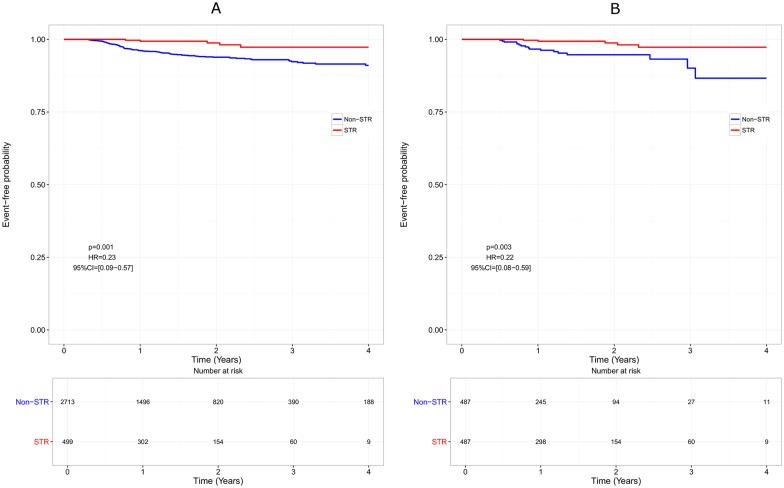
Virological efficacy over time. Virological failure is defined as viral load (VL) > 1000 copies/mL between W16 and W24 or VL > 200 copies/mL after W24.

The median time to treatment discontinuation was shorter in non-STR patients than in STR patients, both in the whole cohort (1.38 years [1.28–1.47] vs 2.70 [2.36–3.50], HR = 0.61, p<0.0001), and in the matched cohort (1.20 years [1.02–1.38] vs 2.70 [2.36–3.50], HR = 0.55, p<0.0001; Fig 1). However, this difference disappeared when censoring ART modification for simplification, both in the whole cohort (HR = 0.97, p = 0.65) and in the matched cohort (HR = 0.90, p = 0.32; Fig 2). The efficacy analysis based on virological response only, indicated a beneficial effect of STRs compared with non-STR regimens with a longer time to virological failure (HR = 0.23, p = 0.001 in the whole cohort and HR = 0.22, p = 0.003 in the matched cohort; Fig 3).

### Tolerance and toxicity over time

**Fig 4:**
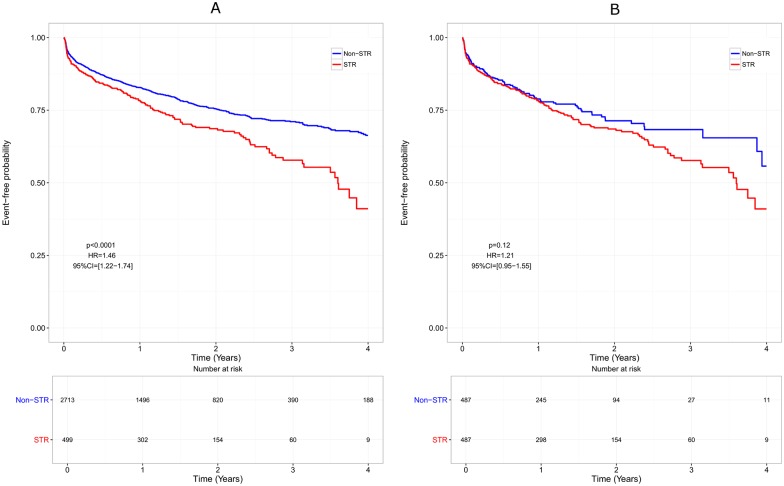
Tolerance over time: Failure is defined as the occurrence of an adverse event anytime during therapy.

Using the whole cohort, time to occurrence of adverse events leading to treatment discontinuation was shorter in patients treated with STRs than in patients treated with non-STRs (HR = 1.46, p<0.0001). This difference was no longer statistically significant in the matched cohort (HR = 1.21, p = 0.12) (Fig 4).

### Subgroup analysis

A total of 499 patients receiving an STR with 2 NRTI + 1 NNRTI (100%) were compared with 872 non-STR patients receiving similar drug components (32.1%). Results are presented in S2 Table. Similar virological efficacy and similar tolerance were observed between STR and non-STR groups.

## Discussion

Based on a cohort of 3212 naïve patients, our study compared the effectiveness and tolerance of STR and non-STR once-daily regimens in real life conditions. When considering treatment failure as a virological failure or any treatment modification over time, a benefit was observed for STRs, which induced less ART modifications than non-STR regimens. However, our results show that this effect is mainly due to treatment simplifications that occurred in almost 28% of patients in the non-STR group (Table 2). Another study based on a different methodology using data from the French National Healthcare Insurance database, also showed that treatment persistence (continuous treatment without interruption or modification) was higher in patients treated with an STR compared with other administration schedules [22]. However, the reasons for treatment modifications could not be determined in this study.

When focusing on virological efficacy only, a benefit of STRs is observed whereas non-STR once-daily regimens seem to be associated with a better tolerance. It should be noticed that the rate of virological failure in both groups of patients is very low (<5% in the matched cohort). Therefore, the benefit of STRs on virological efficacy, if any, may only have a limited clinical impact. Since the difference in tolerance observed on the whole cohort disappears when focusing on the matched cohort (Fig 4), it is reasonable to believe that the difference resulted from different patients’ characteristics.

The Dat’AIDS cohort is a French large multicenter prospective cohort based on data from about 30 HIV centers scattered throughout the country [19] and including data on more than 35,000 HIV patients. This cohort may be considered as representative of real-life clinical practice. Characteristics at baseline showed significant differences between both treatment strategies indicating that STR and non-STR once-daily regimens are associated with different patient profiles. Patients receiving STR were indeed younger, more often male, had higher CD4 and lower HIV RNA level and had less often a CDC stage C suggesting that these patients had somehow less severe infection than patients receiving a non-STR regimen. To reduce these differences, a matched control cohort was selected based upon propensity scores. After matching, the STR and non-STR groups were comparable in regards to baseline characteristics. However, except for the tolerance analysis (Fig 4), no clear-cut difference appeared when analyzing the whole cohort or the matched cohort.

Nowadays, most international guidelines recommend decreasing pill burden to prevent non-adherence to treatment. Some authors have shown that besides a 20% rate of complete non-adherence, 3–13% of patients reported a selective non-adherence (to one or several compounds in a given regimen) hereby stressing the potential benefit of STRs [23]. Lower pill burden has been associated with both better adherence and virological suppression although a meta-analysis reported that adherence, but not virological suppression, was slightly better with once- vs twice-daily regimens [24]. A recent meta-analysis showed that patients on STR were significantly more adherent than patients both on once- and twice-daily multi-tablet regimens and presented a higher rate of viral load suppression at 48 weeks [25]. However, the meta-analysis by Nachega et al. [24] did not contrast STR versus several pills provided once-daily, which was the purpose of the current analysis. In addition, the potential benefit of STRs should be weighed against the higher costs of these newest branded combinations compared with non-STR regimens that may contain generic compounds [18]. Another drawback is that STRs are based on fixed-dose combinations making it impossible to adapt a single compound unless breaking the regimen into several pills. Our study has some limitations. The most important one is the retrospective comparison with absence of randomization. Comparison of baseline characteristics shows that treatment was allocated by choice. STRs were indeed prescribed to patients with a higher chance of favorable outcomes compared with patients receiving non-STR as first-line ART. Because the database used yielded from a clinical cohort rather than an administrative database [22], this bias was limited by creating a matched cohort of non-STR patients who turned to be closely comparable to STR patients. Another limitation is that the proportion of individual drug components differed between STR and non-STR groups. All STR patients were indeed treated with 2 NRTI + 1 NNRTI compared with only 32% of non-STR patients. When comparing these subgroups (S2 Table), no clear-cut benefit of STRs is observed in terms of virological efficacy and tolerance. It is thus likely that the lower tolerance observed in STR patients in the whole cohort is due to the higher proportion of patients receiving efavirenz. Since newer STRs—especially those containing integrase inhibitors—are probably better tolerated [26,27], our results cannot be extrapolated to all different STRs. At the time of the study, TDF/FTC was the only nucleoside combination available as STR and comparison with abacavir/lamivudine combination could not be performed. However, the small difference in virological efficacy between STR and non-STR regimens suggests a limited impact of nucleosides on virological failure. Another possible limitation is that presence of comorbidities is not controlled for in the model. Patients with multiple comorbidities are indeed more likely to receive other non-HIV medications and have thus a higher chance to discontinue therapy because of pharmacological interaction. Comorbidities may also directly influence the choice of ART or limit the choice of treatment: for example, patients with renal diseases are contraindicated to receive tenofovir and may not receive tenofovir-based STR. It is also possible that non-STR were specifically prescribed to patients because of the presence of resistance associated mutation on genotypic results. However, primary resistance to STR compounds was rare in France at the time of the study (<5%) [28]. Finally, we did not measure adherence to therapy. However, a low level of adherence would have primarily affected the virological results [29,30]. Since the rate of virological failure is extremely low in this real life study, we can hypothesized that insufficient adherence is probably not a significant issue at the population level. Indeed, a previous French study observed a mean adherence rate of 89.6% in patients in the STR group (n = 76) compared with 86.4% in the non-STR once-daily group (n = 242) [22]. These results, as well as our study, suggest that both STR and once-daily non STR regimens are associated with high level of adherence in real life and that both options may be specifically useful in patients struggling for treatment adherence.

In conclusion, the median time to treatment discontinuation was shorter in non-STR patients than in STR patients but this difference was driven by ART modification for simplification while a STR cannot yet be simplified further. Virological efficacy seems slightly better with STRs than with non-STR regimens. STRs were prescribed to patients with a higher chance of favorable outcomes than patients receiving non-STR as first-line ART. After accounting for baseline differences, the superiority of STRs over non-STR regimens was mainly driven by treatment simplification, suggesting a preference for STR rather than indicating failing therapy [31]. STRs were associated with a slightly better virological control while non-STR regimens seemed to be associated with a better tolerance profile. If the preference for a lower pill burden is not considered, limited differences could be expected using current OD regimens instead of STRs as first-line ART. Moreover, switching STRs to non STR once-daily regimens using antiretroviral generics could be similarly efficient and more cost-effective. In this context, STR regimens could be useful in patients struggling for treatment adherence [10].

## Supporting Information

S1 Table Detailed antiretroviral treatment among patients receiving multiple tablets once-daily (non-STR) or single tablet regimen (STR). (PDF)

S2 Table Sensitivity analyses comparing Single Tablet Regimens (STR) (n = 499) and non-STR regimens (n = 872) restricted to those based on 2NRTI + 1 NNRTI. “Overall effectiveness” indicates that failure is defined as treatment discontinuation, occurrence of adverse event, or any cause of treatment modification. “Overall effectiveness (simplification censored)” indicates that failure is defined as treatment discontinuation, occurrence of adverse event, or any cause of treatment modification except treatment simplification (censored). “Virological efficacy” indicates that virological failure is defined as viral load (VL) > 1000 copies/mL between W16 and W24 or VL > 200 copies/mL after W24. “Tolerance” indicates that failure is defined as the occurrence of an adverse event anytime during therapy. A Hazard Ratio (HR) <1 is in favor of STR.
(PDF)
