# Supplementary material for: Effectiveness and tolerance of single tablet versus once daily multiple tablet regimens as first-line antiretroviral therapy - Results from a large french multicenter cohort study
Source: PLoS One. 2017 Feb 2;12(2):e0170661. doi: 10.1371/journal.pone.0170661 (PMC5289500; doi:10.1371/journal.pone.0170661)
Supplement: S1 Table — (PDF) [file pone.0170661.s001.pdf]

S1 Table: Detailed antiretroviral treatment among patients receiving multiple tablets once-daily (non-STR) or single tablet regimen (STR)

	Non-STR group (n=2713)	STR group (n=499)
2 NRTI + 1 NNRTI		
Efavirenz	872 (32.1%)	473 (94.8%)
Rilpivirine	0 (0%)	26 (5.2%)
2 NRTI + 1 bPI		
Atazanavir	963 (35.5%)	0 (0%)
Darunavir	878 (32.4%)	0 (0%)
NRTI backbone		
Truvada/emtricitabine	2190 (80.7%)	499 (100%)
Abacavir/lamivudine	523 (19.3%)	0 (0%)

NRTI, nucleoside reverse transcriptase inhibitor; NNRTI, non-nucleoside reverse transcriptase inhibitor; bPI, boosted protease inhibitor; STR, single tablet regimen.
