# Supplementary material for: Effectiveness and tolerance of single tablet versus once daily multiple tablet regimens as first-line antiretroviral therapy - Results from a large french multicenter cohort study
Source: PLoS One. 2017 Feb 2;12(2):e0170661. doi: 10.1371/journal.pone.0170661 (PMC5289500; doi:10.1371/journal.pone.0170661)
Supplement: S2 Table — “Overall effectiveness” indicates that failure is defined as treatment discontinuation, occurrence of adverse event, or any cause of treatment modification. “Overall effectiveness (simplification censored)” indicates that failure is defined as treatment discontinuation, occurrence of adverse event, or any cause of treatment modification except treatment simplification (censored). “Virological efficacy” indicates that virological failure is defined as viral load (VL) > 1000 copies/mL between W16 and W24 or VL > 200 copies/mL after W24. “Tolerance” indicates that failure is defined as the occurrence of an adverse event anytime during therapy. A Hazard Ratio (HR) <1 is in favor of STR. (PDF) [file pone.0170661.s002.pdf]

S2 Table: Sensitivity analyses comparing Single Tablet Regimens (STR) (n=499) and non-STR regimens (n=872) restricted to those based on 2NRTI + 1 NNRTI. "Overall efficacy" indicates that failure is defined as treatment discontinuation, occurrence of adverse event, or any cause of treatment modification. "Overall efficacy (simplification censored)" indicates that failure is defined as treatment discontinuation, occurrence of adverse event, or any cause of treatment modification except treatment simplification (censored). "Virological efficacy" indicates that virological failure is defined as viral load (VL) > 1000 copies/mL between W16 and W24 or VL > 200 copies/mL after W24. "Tolerance" indicates that failure is defined as the occurrence of an adverse event anytime during therapy. A Hazard Ratio (HR) <1 is in favor of STR.

	Whole cohort			After propensity matching		
	HR	95% CI	p	HR	95% CI	p
Overall efficacy	0.38	0.33-0.44	<0.001	0.37	0.32-0.43	<0.001
Overall efficacy (simplification censored)	0.86	0.73-1.02	0.091	0.86	0.72-1.01	0.073
Virological efficacy	1.01	0.83-1.23	0.902	0.95	0.78-1.15	0.597
Tolerance	1.22	0.99-1.50	0.067	1.13	0.91-1.40	0.271

HR, hazard ratio; 95% CI, 95% confidence interval
